# Targeting DNA double strand break repair with hyperthermia and DNA-PKcs inhibition to enhance the effect of radiation treatment

**DOI:** 10.18632/oncotarget.11798

**Published:** 2016-09-01

**Authors:** Bregje van Oorschot, Giovanna Granata, Simone Di Franco, Rosemarie ten Cate, Hans M. Rodermond, Matilde Todaro, Jan Paul Medema, Nicolaas A.P. Franken

**Affiliations:** ^1^ Laboratory for Experimental Oncology and Radiobiology (LEXOR), Center for Experimental Molecular Medicine, Department of Radiation Oncology, Academic Medical Center, Cancer Genomics Center, Amsterdam, The Netherlands; ^2^ Department of Surgical, Oncological and Stomatological Sciences (DICHIRONS), Cellular and Molecular Pathophysiology Laboratory, University of Palermo, Palermo, Italy; ^3^ Biomedical Department of Internal and Specialistic Medicine (DIBIMIS), University of Palermo, Palermo, Italy

**Keywords:** radiation oncology, DNA repair, hyperthermia, double-strand breaks

## Abstract

Radiotherapy is based on the induction of lethal DNA damage, primarily DNA double-strand breaks (DSB). Efficient DSB repair via Non-Homologous End Joining or Homologous Recombination can therefore undermine the efficacy of radiotherapy. By suppressing DNA-DSB repair with hyperthermia (HT) and DNA-PKcs inhibitor NU7441 (DNA-PKcs*i*), we aim to enhance the effect of radiation.

The sensitizing effect of HT for 1 hour at 42°C and DNA-PKcs*i* [1 μM] to radiation treatment was investigated in cervical and breast cancer cells, primary breast cancer sphere cells (BCSCs) enriched for cancer stem cells, and in an *in vivo* human tumor model. A significant radio-enhancement effect was observed for all cell types when DNA-PKcs*i* and HT were applied separately, and when both were combined, HT and DNA-PKcs*i* enhanced radio-sensitivity to an even greater extent. Strikingly, combined treatment resulted in significantly lower survival rates, 2 to 2.5 fold increase in apoptosis, more residual DNA-DSB 6 h post treatment and a G2-phase arrest. In addition, tumor growth analysis *in vivo* showed significant reduction in tumor growth and elevated caspase-3 activity when radiation was combined with HT and DNA-PKcs*i* compared to radiation alone. Importantly, no toxic side effects of HT or DNA-PKcs*i* were found.

In conclusion, inhibiting DNA-DSB repair using HT and DNA-PKcs*i* before radiotherapy leads to enhanced cytotoxicity in cancer cells. This effect was even noticed in the more radio-resistant BCSCs, which are clearly sensitized by combined treatment. Therefore, the addition of HT and DNA-PKcs*i* to conventional radiotherapy is promising and might contribute to more efficient tumor control and patient outcome.

## INTRODUCTION

The underlying mechanism of many anti-cancer treatments, including ionizing radiation, is the induction of lethal DNA double strand breaks (DSB) [1, 2]. The more rapidly dividing tumor cells are thought to be more sensitive to ionizing radiation then healthy cells, and their subsequent DNA damage response less efficient [2]. However, tumor cells can still repair the induced DSB thereby undermining the effectiveness of therapy. Furthermore, some tumor cells are thought to be less sensitive to radiation treatment [3], *i.e.* cancer stem cells, which might resist therapy or repair DNA breaks more efficiently [4]. Therefore, a suggested mechanism to sensitize tumor cells and cancer stem cells to radiation, is the inhibition of DNA-DSB repair proteins [5, 6]. In mammalian cells, DSB are repaired predominantly by non-homologous end joining (NHEJ) or homologous recombination (HR) [7, 8]. A complex cascade of reactions is initiated after a DSB has been induced. ATM kinase and the Mre11/Rad50/NBS1 (MRN) complex are triggered and subsequently the histone protein H2AX is phosphorylated at the DSB sites to γ-H2AX, presenting one of the earliest markers of DSB [9–11]. Other DSB repair proteins, including MDC1, 53BP1 and RAD51 are then attracted to the break ends and, accompanied by γ-H2AX, form ionizing radiation induced foci (IRIF) [12, 13]. After initial recognition, DSB repair can be executed. Failure of repair proteins to form IRIF has been linked to damage response deficiencies [14]. Interestingly, several studies associate the induction and disappearance of γ-H2AX IRIF *in vitro* with treatment response in tumors and normal tissue [15–20]. The higher the number of induced γ-H2AX foci or slower disappearance rate, the more sensitive tumor cells are to radiation treatment. Furthermore, persisting γ-H2AX IRIF in normal cells 24 h after radiation are associated with the development of late severe side effects.

HR requires a homologous DNA sequence to repair the broken strand and therefore is mainly active during the S and G2 phases of the cell cycle when a DNA template is available in the form of a sister chromatid [21]. The major HR factors include Rad51, Rad54, BRCA2 and RPA [22]. Contrarily, NHEJ is active during all phases of the cell cycle as it ligates DNA break ends without requiring a homologous sequence. Therefore NHEJ is thought to be the less accurate form of DSB repair [23]. One of the key proteins in the NHEJ process is DNA-PK. After the induction of a DSB, the KU heterodimer, consisting of the KU70 and KU80 proteins, binds DNA break ends and recruits the DNA-dependent protein kinase catalytic subunit (DNA-PKcs), which leads to the formation of the DNA-PK holo-enzyme [21]. DNA-PK then forms a functional complex with Artemis, which provides nucleolytic processing activity required to prepare DNA ends for ligation [24].

Hyperthermia (HT) is currently being used in the clinic and has proven to be a potent sensitizer of radiotherapy and/or chemotherapy [25, 26]. Krawczyck *et al*. [27] showed that hyperthermia transiently degrades the BRCA2 protein and subsequently prevents the RAD51 protein from accumulating at the break ends. The inactivation of RAD51 and BRCA2 leads to a temporarily inhibition of the HR repair. However, blocking HR repair could lead to a compensated NHEJ DSB repair [6, 28]. Therefore, we want to investigate the inhibition of both HR and NHEJ repair pathways. Here, NHEJ repair was inhibited by the specific DNA-PKcs inhibitor NU7441 (DNA-PKcs*i*) [29, 30]. Results show that a combination of both repair inhibitory modalities clearly enhanced radiosensitivity more than the single treatments did in experimental cell lines, BCSCs as well as in human tumor mouse models.

## RESULTS

### DNA-PKcs*i* and hyperthermia sensitize cancer cells and BCSCs to radiation treatment

**Figure 1:**
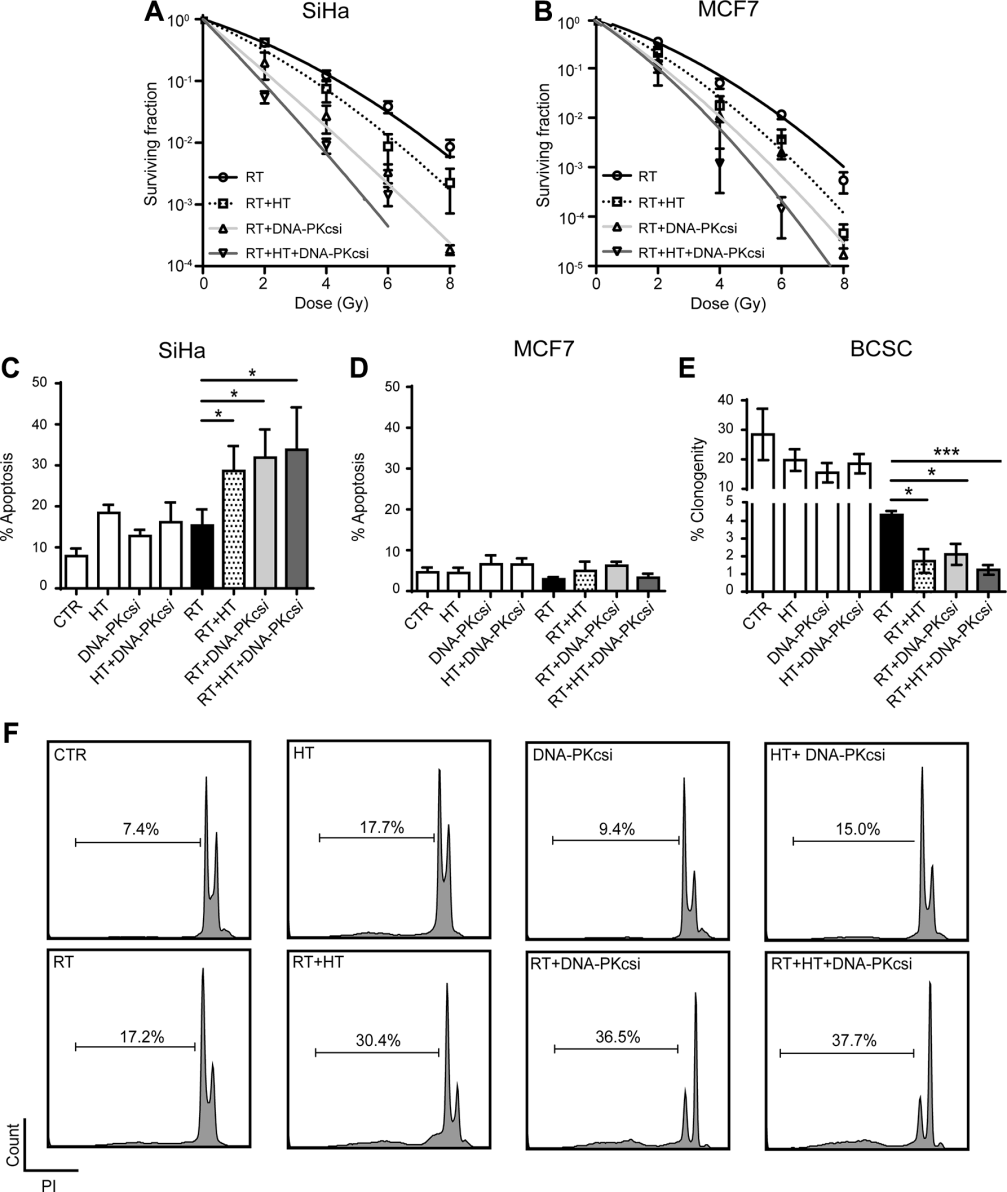
Cancer cells and BCSCs are clearly sensitized to ionizing radiation (RT) by DNA-PKcs inhibition and hyperthermia (HT) (**A**) and (**B**) Clonogenic assay with increasing radiation dose after combined treatment in SiHa (A) and MCF7 (B) cells. Survival curves were established using the linear quadratic regression model, corresponding α and β values can be found in Supplementary Table S1. (**C**) Levels of apoptosis 48 h after different combinations of treatment in SiHa cells. (**D**) Levels of apoptosis in MCF7 cells cannot be detected as they harbor a CASP3 mutation, treated as in (C). (**E**) Limiting dilution analysis for BCSCs treated as in (C). (**F**) Flow cytometer plots presenting results of nicoletti assay in SiHa cells. All experiment were performed at least three times, independently and error bars represent SD, significance is indicated with horizontal lines (*P < 0.05, **P < 0.01, ***P < 0.001).

Clonogenic survival assays were performed to study whether the inhibition of HR in combination with the prevention of NHEJ can lead to a more effective therapy. Results demonstrated a clear radio-enhancement when the cells are treated with either DNA-PKcs*i* or hyperthermia prior to irradiation, indicated by significant lower survival fractions compared to radiation alone in SiHa and MCF7 cells (Figure 1A–1B). This radiosensitizing effect is observed in an even greater extent when a combination of both treatments is used, leading in all assessed cell lines to a significant decrease in clonogenic survival (Supplementary Figure S1A–S1B; SiHa *p <* 0.001; MCF7 *p* = 0.002*;* HeLa *p <* 0.001 and T47D *p* = 0.007). In Supplementary Table S1, values of the parameters of the Linear Quadratic model are presented. Hyperthermia, DNA-PKcs*i* and the combination of both, resulted in a higher induction of unrepairable DNA damage in cells when compared to irradiation alone, indicated by the higher α-values after combined treatment strategies. Furthermore, the reduced clonogenic survival is confirmed by increasing levels of apoptosis after combination treatments (Figure 1C and 1F, Supplementary Figure S1C–S1D). As can be depicted from Figure 1C, radiation alone does not induce apoptosis in SiHa cells, but when combined with DNA-PKcs*i* and HT, a strong apoptotic response is detected. As MCF7 cells are deficient of caspase 3, no levels of apoptosis could be measured when using the Nicoletti assay in this cell line (Figure 1D) [31]. Limiting dilution analysis with BCSCs that are enriched for cancer stem cells (Figure 1E) showed a significant decrease in clonogenic growth after radiation, and this decrease is further enhanced by HT or DNA-PKcs*i*. Strikingly, the combination of radiation with HT and DNA-PKcs*i* resulted in a 3-fold reduction with regard to clonogenic capacity when compared to radiation alone (*p* = 0.001).

### Delayed disappearance of DNA-DSB IRIF after HT and DNA-PKcs*i*

**Figure 2:**
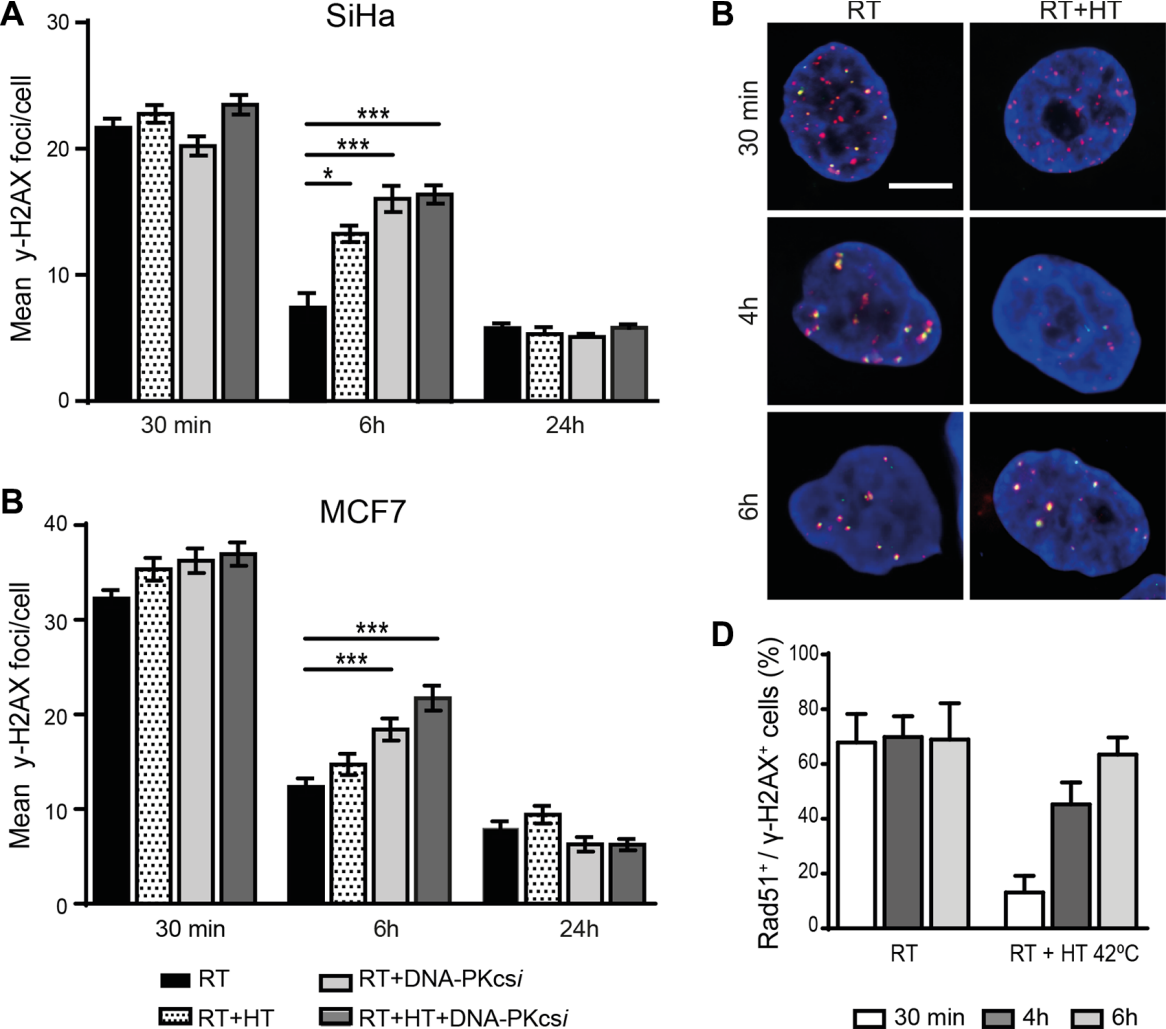
Persisting ionizing radiation induced foci (IRIF) in SiHa and MCF7 cells after radiation treatment combined with DNA-PKcsi and HT (**A**–**B**) quantification of γ-H2AX IRIF in SiHa (A) and MCF7 (B) cells. (**C**–**D**) Visualization (C) and quantification (D) of the significant decrease in co-localization of Rad51 (green) and y-H2AX foci (pink) directly after treatment with hyperthermia in SiHa cells. For each radiation condition at least 100 cells per experiment (*n* = 3) are counted, error bars are ± SEM and significance is indicated with horizontal lines (*P < 0.05, **P < 0.01, ***P < 0.001). Bar is 5 μm.

In order to examine whether the radiosensitizing effect of HT and DNA-PKcs*i* are caused by hampered DNA-DSB repair, numbers of γ-H2AX IRIF were scored at several time points post treatment (Figure 2A–2C, Supplementary Figure S2 and Supplementary Table S2). The addition of HT and DNA-PKcs*i* did not influence the initial induction of DNA-DSB after radiation. Similar numbers of γ-H2AX foci per cell are detected for all cell lines 30 min after different treatments, indicating that the amount of radiation-induced damage is the same in all conditions. However, 6 h post treatment, significantly higher numbers of foci were found after RT combined with DNA-PKcs*i* and/or HT when compared to RT alone. The average numbers of DNA-DSB per cell also highlighted the distinct effect of the triple treatment strategy compared to the double (RT with either HT or DNA-PKcs*i*). Nevertheless, persisting DNA-DSB seemed to be repaired later on, as no differences in IRIF numbers are detected 24 h post treatment. Only for HeLa cells, DNA-DSB repair seemed hindered for a longer period as even after 24 h a significant higher numbers of foci were detected in the triple treatment compared to RT alone (Supplementary Figure S2A). Mechanistically, a temporarily decrease of Rad51 accumulation at the site of γ-H2AX IRIF (Supplementary Figure S3) after hyperthermia treatment was indeed observed, and fully restored after 6 h.

### Radio-sensitization after HT and DNA-PKcs*i* is accompanied by a G2-phase arrest

**Figure 3:**
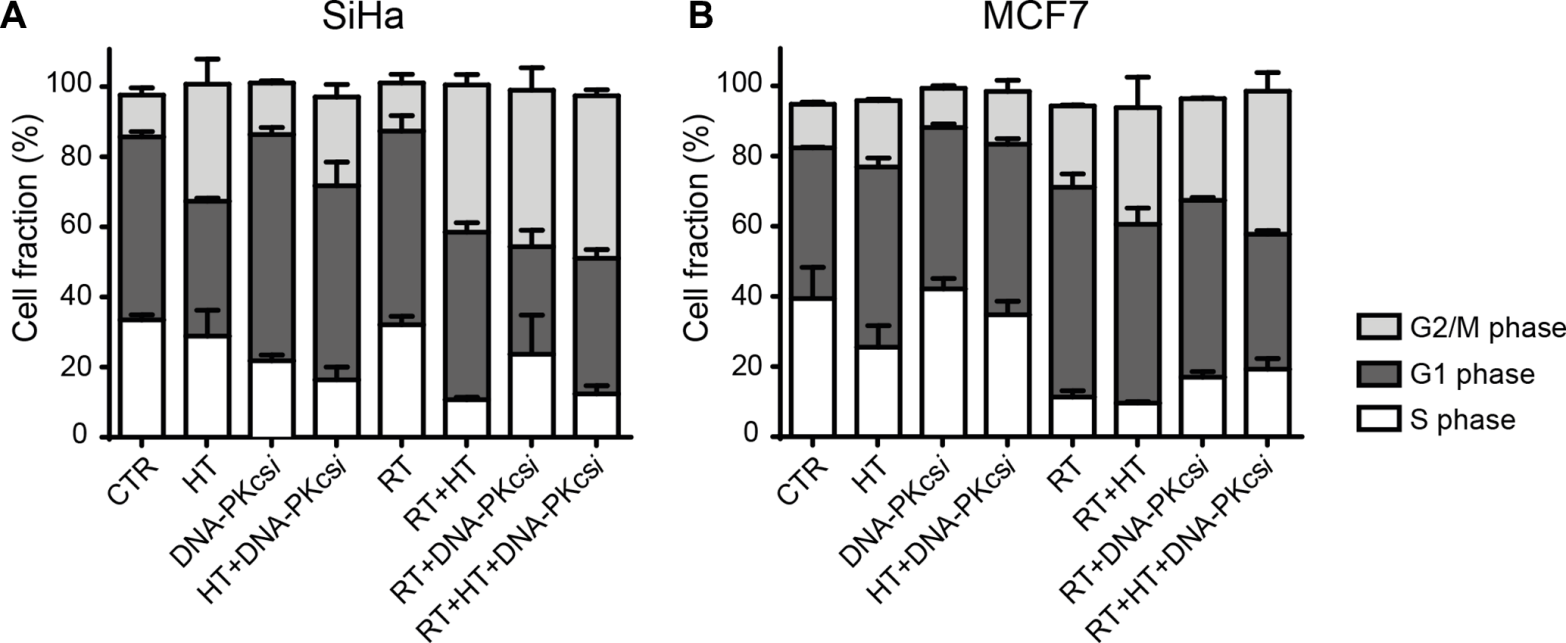
Radiosensitization of DNA-PKcsi and HT is accompanied with an induced G2/M arrest 16 h after radiation (**A**–**B**) Cell cycle analysis of SiHa (A) and MCF7 (B) cells treated with radiation, hyperthermia and/or DNA-PKcs*i*. As can be depicted, HT alone also affected SiHa cells in their cell cycle. For MCF7 this effect was not detected. Experiments are performed at least three times, error bars represent SD.

The effect of combined treatment modalities on cell cycle distribution was measured 16 h post treatment. In general, ionizing radiation induced a cell cycle progression arrest in both the G1 (SiHa, MCF7) and G2 (HeLa, T47D) phase depending on cell type. Interestingly, the combination of DNA-PKcs***i*** and HT with radiation resulted in a marked accumulation of cells in G2 phase for all cell types (Figure 3A–3B and Supplementary Figure S4).

### Tumor growth delay *in vivo* and higher levels of apoptosis in xenografts

**Figure 4:**
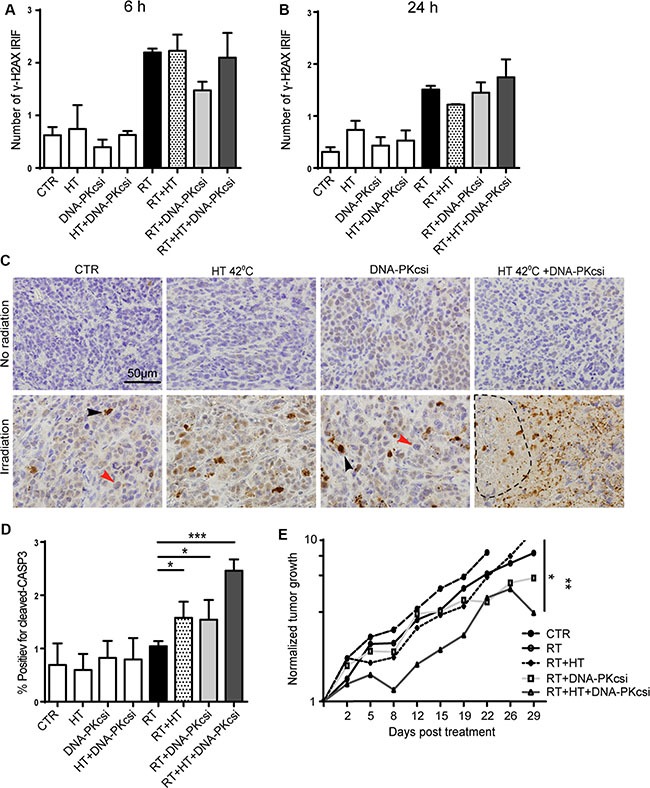
Higher levels of apoptosis and clear tumor growth delay *in vivo* after combination treatments (**A**–**B**) Induction of γ-H2AX IRIF in xenografts collected 6 h (A) or 24 h (B) post treatment. (**C**) Immunohistochemically detection of Cleaved Caspase-3 48 h after treatment to measure apoptosis *in vivo*, induced levels of apoptosis in xenografts treated with radiation in combination with DNA-PKcs*i* and HT compared to radiation alone. Necrotic areas were observed in xenograft of triple treatment. (**D**) Quantification of C-CASP3 staining in xenografts, as in (C). Three xenografts per treatment condition were analyzed for Caspase 3 and γ-H2AX immunostainings. (**E**) Tumor growth delay analysis after triple treatment compared to radiation alone. Tumor sizes were biweekly measured and normalized to initial size, every treatment group consisted of 6 mice.

Further confirmation of the sensitizing effect that the combined treatment modalities have, was investigated in an *in vivo* tumor model consisting of SiHa cells in athymic mice. DNA-DSB and apoptotic markers were analysed shortly after treatment, while tumor growth was observed for approximately 30 days. The DNA-DSB induction was measured by scoring γ-H2AX IRIFs in xenografts 6 h and 24 h post treatment. Similar numbers of γ-H2AX foci were detected for all irradiated xenografts 6 h post treatment whereas after 24 h, numbers only slightly reduced. No correlations were found with *in vitro* IRIF analysis or hindered DNA-DSB repair after DNA-PKcs*i* or HT treatment (Figure 4A–4B). Contrarily, levels of apoptosis *in vivo* were induced to a similar extent as measured in the cell lines by the combined treatment. Apoptosis was measured *in vivo* by detecting cleaved caspase-3 48 h after treatment. Significantly higher levels of cleaved caspase-3 were detected in xenografts that received radiation combined with HT and DNA-PKcs*i* (*p <* 0.0001) See Figure 4C–4D). Interestingly, necrotic regions were only observed in the xenografts of the triple treatment modality. Furthermore, the triple treatment, ionizing radiation combined with HT and DNA-PKcs*i*, resulted in a significant tumor growth delay compared to ionizing radiation alone (*p* = 0.004) or ionizing radiation combined with only HT (*p* = 0.03). In Figure 4E and Supplementary Figure S5, normalized tumor growth curves are presented with regard to all treatment groups.

## DISCUSSION

This study investigated the additional effects of DNA-DSB repair inhibitors when added to conventional radiotherapy. Our results show that both HT and DNA-PKcs*i* enhance the effect of radiation treatment significantly, especially when both modalities are combined. Lower surviving fractions, more residual DNA damage and a G2-phase arrest, were detected after combined treatment in all examined cervical – and breast cancer cell lines. Interestingly, the assumed radio-resistant BCSCs were also affected to a higher degree after a combined treatment strategy as compared to radiation alone. Furthermore, *in vivo* results verify the importance of adding both HT and DNA-PKcs*i* to conventional radiation treatment. The highest level of apoptosis was detected and tumor growth was most delayed after the triple treatment strategy.

Results of the clonogenic survival assay showed that the inhibition of both DNA-DSB repair mechanisms has a large radio-sensitizing effect. The value of the linear parameter α, increased with the different treatments being applied (HT, DNA-PKcs*i* and combined treatment respectively) and corresponds with higher levels of cell reproductive death in the lower dose regions. Subsequently, the value of the quadratic parameter β, dropped with each treatment and almost no functional DNA DSB repair was observed when both repair pathways were inhibited, as the β-value reached zero.

In all cell lines, more persisting DNA-DSB were detected at 6 h, after applying the combined treatment modalities as compared to radiation treatment alone. At 24 h after treatment, the numbers of γ-H2AX foci were reduced to numbers found in untreated, control samples, indicating that cells did eventually repair their DNA-DSB breaks. Thus, the addition of HT and DNA-PKcs to radiation resulted in a slower loss of foci, e.g. slower repair rate, rather than no repair in the cancer cells that survived treatment. This can be explained by the fact that the effect of hyperthermia and NU7441 are only temporary. Foci results obtained in this study, showed that Rad51 was detected at the sites of DSB again 6 hours after HT treatment (Supplementary Figure S3D), indicating active HR repair. This is congruent with other studies, which have shown that hyperthermia degrades BRCA2 only for a few hours [26, 27]. In addition, Zhao et al. [29] showed that the required concentration of NU7441 was only maintained *in vitro* for up to 4 hours, meaning active NHEJ could occur again after 4 hours.

Furthermore, cell cycle analysis revealed an induced G2/M phase arrest after radiation treatment combined with HT and DNA-PKcs*i*. This is in line with the results of other studies [29, 30, 32], and might be explained by activation of other DNA damage pathways in the absence of HR and NHEJ. For example, an activated ATR/Chk1 pathway is related to a profound accumulation in G2 phase in response to RT [33]. In addition, back-up End Joining (B-EJ) processes are also thought to benefit from a G2 arrest [34], hence allowing more time to repair DSB.

Several studies examined the sensitizing effect of DNA-PKcs*i* [29, 30, 32] and HT [26, 27] separately, but the combination of both repair inhibitors, has not yet been tested. As previously mentioned, blocking one repair pathway is thought to lead to the compensation of the other repair pathway [35]. Therefore, inhibiting both at the same time, leads to a more complete and pronounced radio-sensitization. *In vitro* and *in vivo* results obtained in this study showed the largest sensitizing effect when both repair mechanisms were inhibited. Especially tumor growth delay and xenografts analysis elucidated the distinct effect of radiation combined with HT and DNA-PKcs*i*. Furthermore, the possibility that HT and DNA-PKcs*i* could sensitize BCSCs to radiation treatment, is interesting and should be further investigated. The BCSCs are enriched for cancer stem cells, whichare associated with radio-resistance and poor survival [36], and are thought to have a highly active DNA damage response [37]. Therefore, the inhibition of DSB repair mechanisms by HT and DNA-PKcs*i* might have an augmented effect in the cancer stem cell population of the tumor, leading to a better treatment.

In conclusion, the results obtained in this study elucidate the use of HT and DNA-PKcs inhibition together with radiation treatment. The combination treatment magnifies the effect of conventional radiotherapy and is promising for clinical use.

## MATERIALS AND METHODS

### Culture of cancer cells

Human cervical cancer cells: HeLa and SiHa and human breast cancer cells: MCF7 and T47D, were all obtained from the American Type Culture Collection (ATCC). [38] HeLa and SiHa cells were routinely cultured in Eagle's Minimum Essential medium (EMEM, Gibco-brl technologies), MCF7 cells in Dulbecco's modified Eagles medium (DMEM), and T47D cells in Iscove's Modified Dulbecco's medium (IMDM). They were all supplemented with 8% fetal bovine serum and 100 U/ml antibiotic penicillin, streptomycin and 1 mM glutamine (PSG) at 37°C in a 5% CO_2_ humidified chamber.

### Isolation, culture and characterization of primary breast cancer sphere cells

Primary human breast cancer sphere cells (BCSC) were obtained through mechanical and enzymatical digestion of breast cancer tissues, collected at the Department of Surgical, Oncological and Stomatological Sciences, in accordance with the ethical standards of the University of Palermo institutional committee, as previously described [38]. BCSCs were cultured in serum-free DMEM/F12 medium, supplemented with 2% B27 (50 ×, Gibco), basic fibroblast growth factor (bFGF; 10 ng/mL) and EGF (20 ng/mL) in ultra-low attachment flask (Corning). In these culture conditions the breast cancer cells grow as aggregates conventionally defined as “spheres”. In order to assess the presence of cancer stem cells in our BCSC population, BCSCs were characterized in terms of ALDH1 activity, and then subcutaneously injected in NOD/SCID mice to test their ability to form tumor xenografts [39].

### Irradiation, hyperthermia and DNAPKcs*i* treatment

Radiation treatment was performed with γ-irradiation using a ^137^Cs source at a dose rate of about 0.5 Gy/min. Levels of apoptosis and cell cycle distribution were measured after 4 Gy irradiation, numbers of IRIF were detected after 1 Gy, and for clonogenic assay survival analysis cells were irradiated with 0, 2, 4, 6, and 8 Gy. Hyperthermia treatment was performed by incubating cells at 42°C for 1 h in a thermostatically controlled water bath with additional CO_2_. DNA-PKcs was inhibited using the specific inhibitor NU7441, also known as KU-57788 (Selleckchem). NU7441 was dissolved in DMSO as 10 mM stock, further diluted in PBS to 1mM and added to culture medium at a final concentration of 1 μM. DNA-PK activity was measured in whole cell lysates from SiHa cells using the promega SignaTECT^®^ DNA dependent protein kinase assay system, according to manufacturer's protocol (Supplementary Figure S3C).

### *In vivo* tumor model and xenografts

Human cervical SiHa cancer cells were injected into the right hind leg of Athymic mice. Approximately 4 weeks after administration, tumor volumes of 100 mm3 were reached and mice were randomly divided into 8 groups (*n* = 6), all utilizing different treatment combinations and controls. DNA-PKcs inhibitor NU7441 was dissolved in 40% PEG400/Saline and injected i.p. (10 mg/kg) [29] for 4 days before start of HT and RT treatment. For hyperthermia, a water bath system was used were only the right hind leg was treated for 1 h at 42°C. HT was applied only on the first day of treatment. Mice were cooled to prevent an increase of the body core temperature, and anesthetized with a mixture of 2.5% isoflurane and oxygen. Radiation treatment was executed for 4 days with a daily dose of 3Gy using an X-ray RS320 Research cabinet (X-Strahl, 210 kV, 15 mA and 0.5 mmCu filter).

For tumor growth delay analyses, tumor volumes were measured twice a week and mice were sacrificed when tumor volumes reached the size over 1000 mm^3^. Levels of CASP3 and γ-H2AX were detected in xenografts of mice sacrificed 6 h, 24 h or 48 h after treatment. Per treatment condition, 3 xenografts were analyzed by immunohistochemistry. Animal experiments were approved by the animal welfare committee of the Academic Medical Center (AMC) as required by Dutch law LEX143.

### Clonogenic assay

Adhering cells were plated in appropriate cell numbers in 6-well macroplates prior to treatment. After attachment, DNA-PKcs*i* NU7441 or DMSO only (mock treatment) was added and cells were treated with hyperthermia for 1 h. Immediately after hyperthermia, cells were irradiated with doses up to 8 Gy. Cells were incubated for 10 days to form colonies. After the ten day period, surviving colonies were fixated and stained with glutaraldehyde-crystal violet solution and counted manually.

Spheroid BCSC cultures were dissociated and FACS deposited using FACSaria (BD Biosciences) in a limiting dilution manner at 5, 10, 25, 50, 100 and 200 cells per well in ultra-low 96-well plates (Corning). After sorting, DNA-PKcs*i* was added to medium and plates were subjected to HT and RT. Clonal frequency was evaluated with the Extreme Limiting Dilution Analysis ‘limdil’ function as described [40].

### Immunohistochemistry *in vitro* and *in vivo*

Detection and scoring of immunofluorescence γ-H2AX and Rad51 in cell lines, was performed as previously described [15, 27]. Xenografts were fixated in 3.6% paraformaldehyde (Aurion) and embedded in paraffin. Sections of 4 μm were prepared for detection of both cleaved-Caspase3 and γ-H2AX and heat-induced antigen retrieval was performed at pH 6. CASP3 sections continued with peroxidase blocking for 20 min and serum blocking using Ultra-V (Immunologic) for 5 min. Primary antibody Cleaved-Caspase3 (anti rabbit, Cell Signaling) was applied 1:200 overnight at 4°C. Thereafter, sections were incubated with Powervision Poly-HRP-GAM/R/R IgG (Immunologic) for 30 min and PowerDAB (Immunologic) for 1–2 min, counterstained with haematoxylin (Fluka) and mounted with pertex.

For γ-H2AX, sections were blocked after antigen retrieval in PBTB: PBS containing 0.1% Tween20 and 2% Bovine Serum Albumin (BSA) and incubated with primary antibody mouse monoclonal anti- γ-H2AX (Millipore) for 90 min (1:100 in PBTB) at room temperature. Secondary antibody goat anti-mouse Cy3 (Jackson Immunoresearch) was applied for 60 min, and DAPI (Sigma-Aldrich) was used as counterstain. After washing, sections were embedded in Vectashield and analysed by microscopy.

### Cell cycle analyses

Cell cycle analysis was carried out by flow cytometry using Bromodeoxyuridine (BrdU) and propidium iodide (PI) staining. BrdU (10 μM) was administered to cell cultures at 16 h after treatment. After 1 h at 37°C, cells were harvested and fixed overnight in 70% ethanol in PBS. Fixed cells were centrifuged (1 min, 2200 RPM), resuspended in 1ml pepsin-HCL (0.4 mg/ml 0.1N HCL), and incubated for 30 min. PBT (PBS with 0.05% Tween20) was added while vortexing, samples were centrifuged and incubated for 30 min in 1 ml 2N HCL at 37°C. After washing with PBTb (PBT with 20 mg/ml BSA), the pellet was resuspended in 100 μl rat anti-BrdU (Harlan Seralab) diluted 1:100 in PBTb for 60 min at 37°C. For secondary antibody step, cells were washed with PBTg (PBT with 1% v/v normal goat serum (DAKO)) followed by 60 min incubation at 37°C with 0.1 ml fluorescein conjugated goat anti-rat IgG (Jackson Immunoresearch) diluted 1:100 in PBTg. PI was added to a final concentration of 20 μg/ml in PBS and samples were stored at 4°C before flowcytometric (FACS Canto, BD Biosciences) analysis.

### Apoptosis analysis

The Nicoletti assay [41] was used to study apoptosis in adhering cell lines after different treatments. Cells were harvested 48 h post treatment and resuspended in nicoletti buffer (0.1% w/v Sodium Citrate, 0.1% v/v Triton-X in ddH_2_O, pH 7,4) and analysed with flow cytometry (FACS Canto). All experiments were carried out in triplicates, independently from each other.

### Statistical analysis

All experiments were performed at least 3 times, independently, and results are shown as mean ± SD. Survival curves were analyzed using SPSS (Chicago) statistical software by means of fit of data by weighted linear regression, according to the linear-quadratic formula: S(D)/S(0) = exp−(αD + βD^2^) [42, 43]. For γ-H2AX and Rad51 foci detection, at least 100 cells per condition per experiment were scored and the data is presented as the mean ± standard error (SEM). GraphPad Prism 6 was used to perform ANOVA analysis, followed by unpaired Student *t-test* (two tails) for comparison of independent treatments. Significant *P* values are given, * indicates *P <* 0.05, ** indicate *P <* 0.01 and *** indicate *P <* 0.001. ns indicates non statistically significant.

## SUPPLEMENTARY MATERIALS FIGURES AND TABLES


