# Supplementary material for: Targeting DNA double strand break repair with hyperthermia and DNA-PKcs inhibition to enhance the effect of radiation treatment
Source: Oncotarget. 2016 Sep 1;7(40):65504–13. doi: 10.18632/oncotarget.11798 (PMC5323171; doi:10.18632/oncotarget.11798)
Supplement: Supplementary file 1 [file oncotarget-07-65504-s001.pdf]

Targeting DNA double strand break repair with hyperthermia and DNA-PKcs inhibition to enhance the effect of radiation treatment

Supplementary Materials

Supplementary Table S1: Values of the LQ parameters α and β, α- and β- enhancement factor of the assessed cancer cell lines

Cell line	Treatments	α (Gy-1)	β (Gy-2)	α-enhance factor	β-enhance factor	*p*-value (α)
SiHa	RT	0.2 ± 0.04	0.04 ± 0.01			
	RT + HT 42°C	0.5 ± 0.07	0.04 ± 0.01	3.1 ± 0.8	0.9 ± 0.4	0.003
	RT + DNA-PKcs*i*	1.0 ± 0.11	0.02 ± 0.03	5.7 ± 1.4	0.4 ± 0.7	< 0.001
	RT + HT + DNA-PKcs*i*	1.6 ± 0.02	0.07 ± 0.02	9.3 ± 2.1	1.8 ± 0.6	< 0.001
HeLa	RT	0.35 ± 0.07	0.05 ± 0.01			
	RT + HT 42°C	0.49 ± 0.06	0.06 ± 0.01	1.4 ± 0.3	1.2 ± 0.3	0.058
	RT + DNA-PKcs*i*	0.41 ± 0.07	0.06 ± 0.01	1.2 ± 0.3	1.2 ± 0.3	0.351
	RT + HT + DNA-PKcs*i*	0.87 ± 0.06	0.02 ± 0.01	2.5 ± 0.5	0.4 ± 0.2	< 0.001
MCF7	RT	0.5 ± 0.04	0.04 ± 0.01			
	RT + HT 42°C	0.7 ± 0.10	0.04 ± 0.02	1.5 ± 0.2	1.0±0.6	0.032
	RT + DNA-PKcs*i*	1.0 ± 0.12	0.02 ± 0.03	2.1 ± 0.3	1.3±1.0	0.002
	RT + HT + DNA-PKcs*i*	1.0 ± 0.12	0.05 ± 0.04	2.1 ± 0.3	2.2±0.6	0.002
T47D	RT	0.2 ± 0.05	0.07 ± 0.01			
	RT + HT 42°C	0.2 ± 0.06	0.10 ± 0.01	1.2 ± 0.4	1.4 ± 0.3	1.000
	RT + DNA-PKcs*i*	0.1 ± 0.07	0.12 ± 0.02	0.4 ± 0.4	1.7 ± 0.3	0.114
	RT + HT + DNA-PKcs*i*	0.5 ± 0.09	0.11 ± 0.03	2.6 ± 0.8	1.6 ± 0.4	0.007

Clonogenic analysis revealed differences in sensitivity between the assessed cell lines: SiHA and MCF7 are more sensitive to DNA-PKcsi than HT treatment, in contrast to HeLa cells which have a more sensitive response to HT treatment. T47D are not sensitized by either of the two repair inhibitors alone with radiation. However, the combination of both HT and DNA-PKcsi to radiotherapy resulted for all cells in the highest a value and a-enhancement factor, indicating the highest levels of reproductive death is induced after the triple treatment compared to radiotherapy alone or combined with only HT or DNA-PKcs*i*.

Supplementary Table S2: Mean number of γ-H2AX IRIF in untreated, control samples for all cell lines fixed at 30 min, 6 h and 24 h post treatment

Cell line	Condition	30 min	6 h	24 h
SiHa	Untreated	3.1 ± 0.6	3.6 ± 0.5	4.9 ± 0.5
	HT 42°C	2.9 ± 0.3	5.6 ± 0.8	5.7 ± 0.7
	DNA-PKcs*i*	2.8 ± 0.4	5.0 ± 0.6	5.0 ± 0.6
	HT42°C + DNA-PKcs*i*	3.4 ± 0.6	4.5 ± 0.5	5.1 ± 0.6
HeLa	Untreated	9.5 ± 0.9	10.8 ± 0.9	7.4 ± 0.6
	HT 42°C	12.4 ± 1.0	10.1 ± 0.8	6.2 ± 0.5
	DNA-PKcs*i*	9.2 ± 0.7	12.8 ± 1.0	8.5 ± 0.6
	HT42°C + DNA-PKcs*i*	11.5 ± 0.9	10.2 ± 0.7	7.5 ± 0.6
MCF7	Untreated	5.8 ± 0.5	7.9 ± 0.8	7.0 ± 0.7
	HT 42°C	8.9 ± 0.9	6.8 ± 0.7	9.5 ± 0.9
	DNA-PKcs*i*	5.2 ± 0.5	9.1 ± 1.1	8.4 ± 0.8
	HT42°C + DNA-PKcs*i*	8.5 ± 0.8	8.2 ± 0.8	7.9 ± 0.8
T47D	Untreated	13.3 ± 0.9	13.1 ± 0.9	13.0 ± 0.8
	HT 42°C	15.0 ± 0.9	13.5 ± 0.8	13.5 ± 0.8
	DNA-PKcs*i*	16.1 ± 1.1	14.9 ± 0.8	14.8 ± 0.9
	HT42°C + DNA-PKcs*i*	16.7 ± 0.9	16.1 ± 0.9	13.0 ± 0.9
BCSC	Untreated	5.7 ± 0.7	7.6 ± 0.1	6.9 ± 0.3
	HT 42°C	7.1 ± 0.1	7.6 ± 0.8	7.0 ± 0.3
	DNA-PKcs*i*	5.6 ± 0.6	7.2 ± 0.1	6.6 ± 0.4
	HT42°C + DNA-PKcs*i*	7.2 ± 0.1	7.5 ± 0.2	6.9 ± 0.3

As can be depicted from the table, average background numbers are different among cell lines. However, for all cell lines HT and DNA-PKcs inhibitor did not increase or influence the number of γ-H2AX IRIF compared to their specific untreated sample. At least 100 cells per condition were counted, in three independent experiments together with the irradiated conditions shown in the manuscript. Error bars represent SEM.

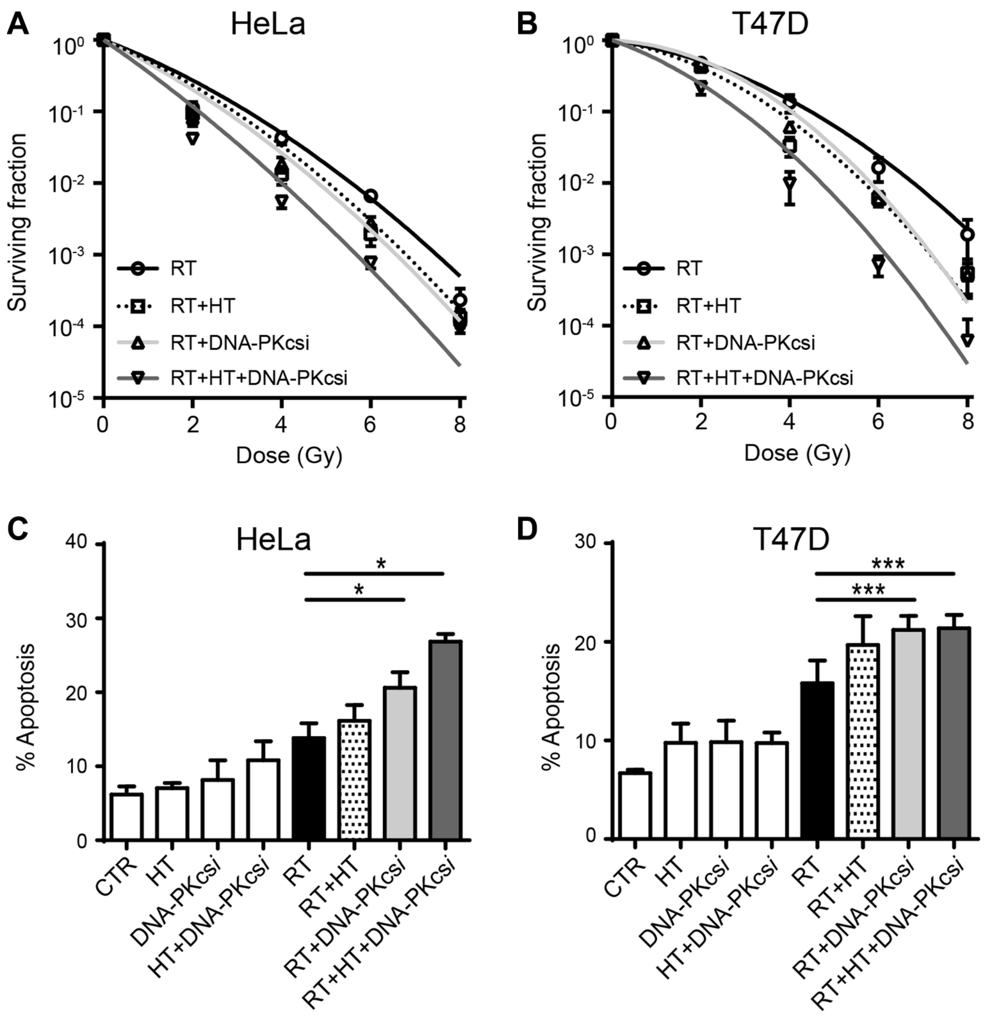

Supplementary Figure S1: Clonogenic survival analysis and apoptosis levels after different treatment combinations: clear radiosensitization after triple treatment. (**A–B**) Clonogenic survival analysis for HeLa (A) and T47D (B) cells. (**C–D**) Nicoletti assay performed on HeLa (C) and T47D (D) cells. All experiment were performed at least three times, independently and error bars represent SD.

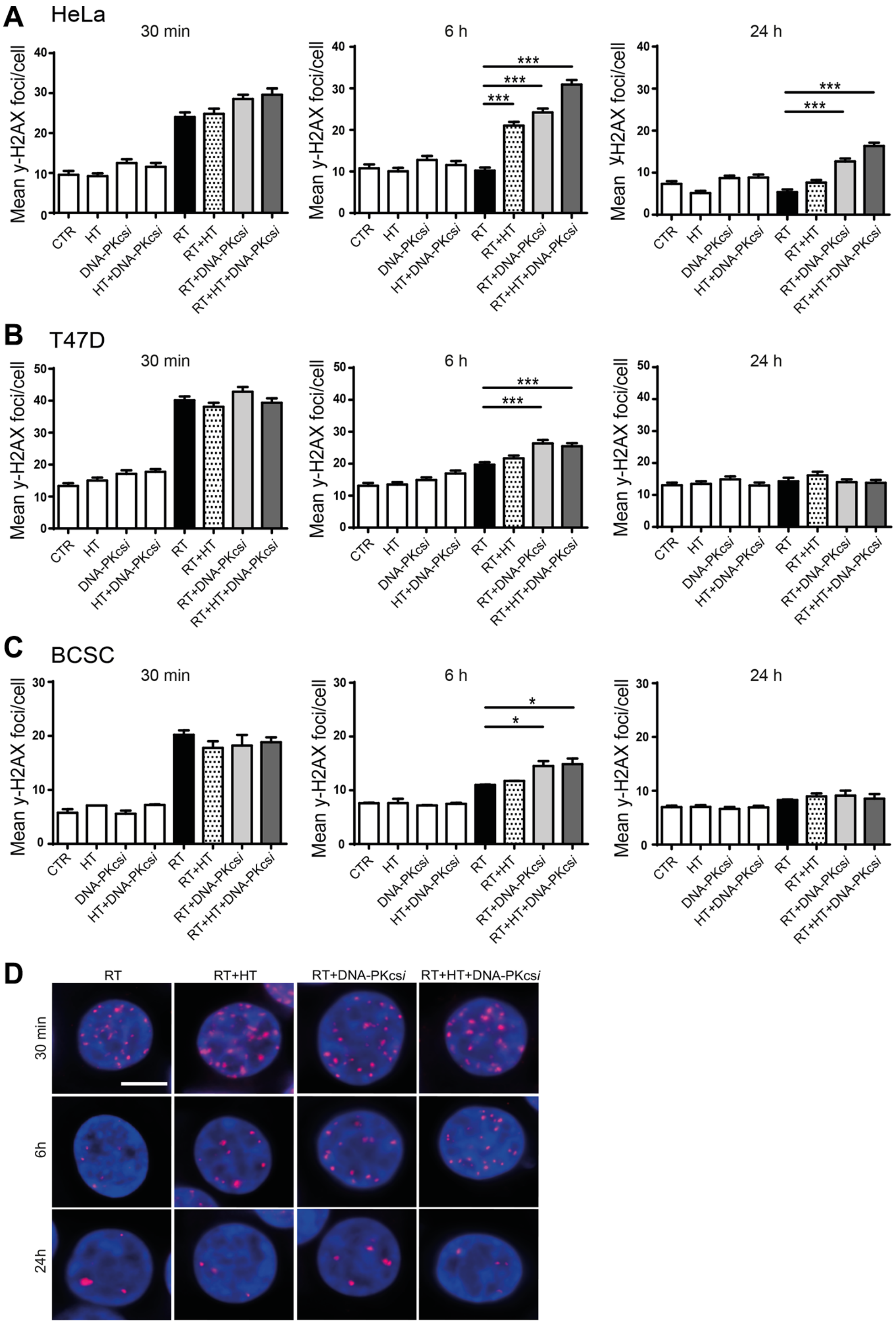

Supplementary Figure S2: More residual DNA-DSBs after radiation treatment combined with HT and DNA-PKcsi indicate for less efficient DNA repair. (**A**–**C**) Results of y-H2AX IRIF analysis for HeLa (A), T47D (B) and BCSCs (**C**) at 30 min, 6 h or 24 h post treatment. (**D**) Visualization of γ-H2AX IRIF 30 min, 6 h, and 24 h after radiation treatment (1 Gy), HT 42°C and DNA-PKcsi [1 μm] in SiHa cells.

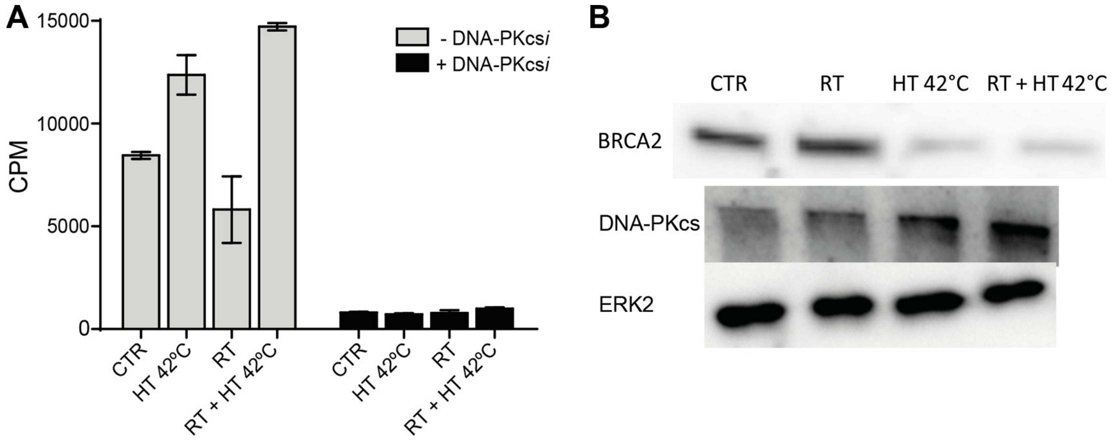

Supplementary Figure S3: Specificity of HT treatment and NU7441. (**A**) DNA-PKcs activity is blocked in presence of DNA-PKcs inhibitor NU7441. Hyperthermia doesn't affect DNA-PKcs activity. (**B**) WB analysis of BRCA2 and DNA-PKcs elucidating the effect of HT treatment on HR pathway regulation

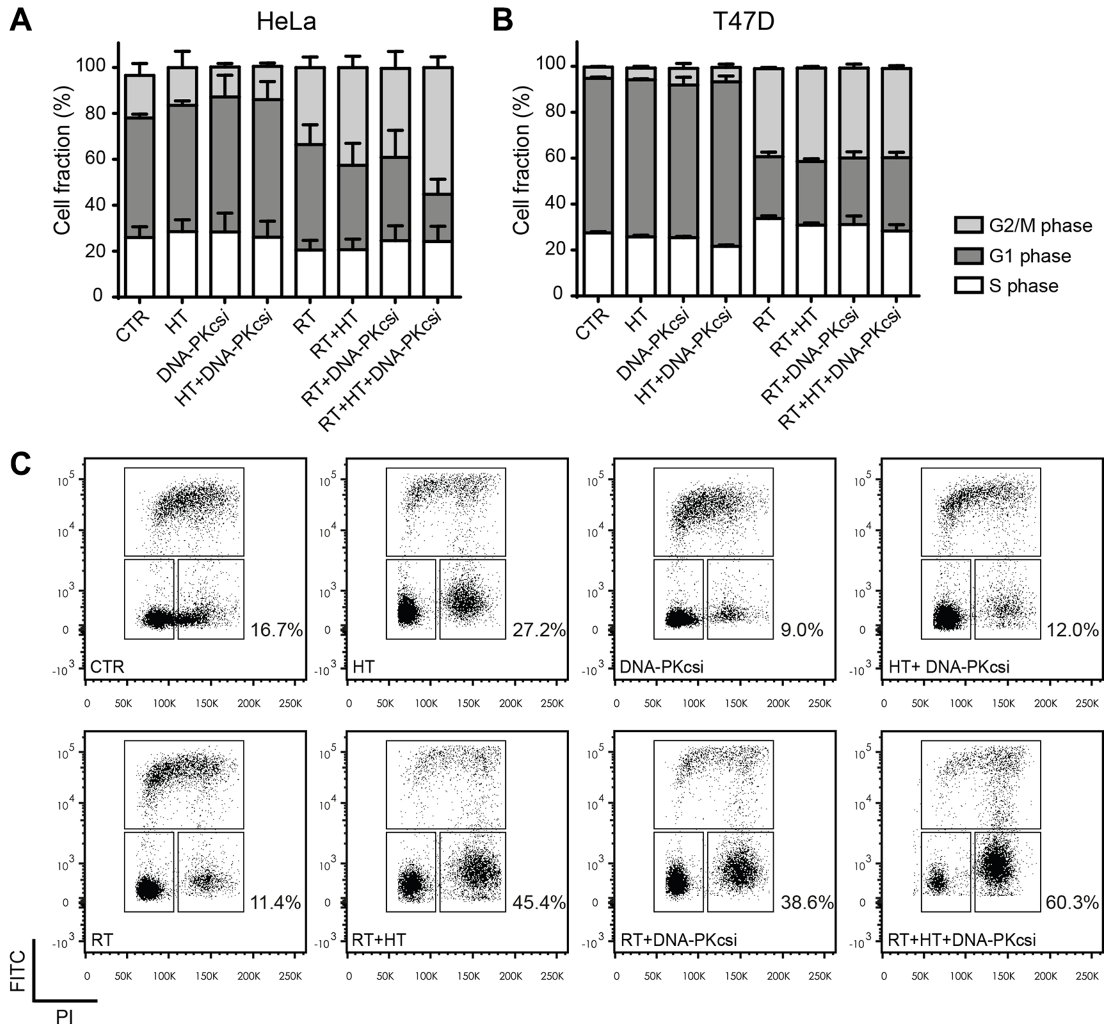

Supplementary Figure S4: Cell cycle distributions in untreated and treated HeLa (A) and T47D (B) cells. Radiosensitization by HT and DNA-PKcsi. Experiments are performed at least three times, error bars represent SD. (**C**) Flow cytometry plots of cell cycle distributions measured by the incorporation of bromodeoxyuridine (BrdU) in SiHa cells.

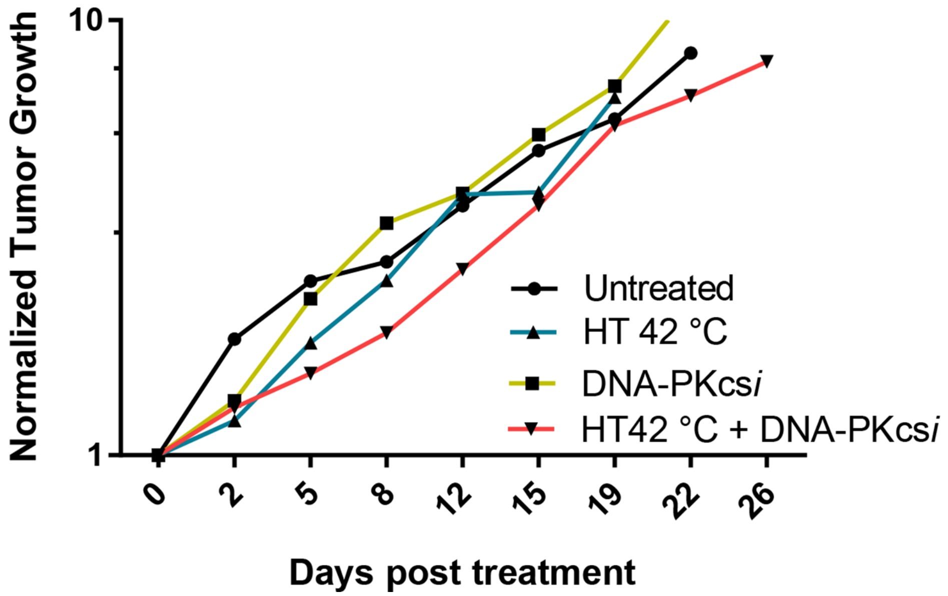

Supplementary Figure S5: Tumor growth delay *in vivo* in untreated andnon-irradiated control conditions. No growth delay between the different conditions could be observed.
